# Psycho-social problems of adolescents with sickle-cell anaemia in Ekiti State, Nigeria

**DOI:** 10.4314/ahs.v21i2.37

**Published:** 2021-06

**Authors:** Lateef Omotosho Adegboyega

**Affiliations:** Department of Counsellor Education, Faculty of Education, University of Ilorin, Ilorin, Nigeria

**Keywords:** Psycho-social problems, sickle-cell anaemia, adolescents

## Abstract

**Background:**

Sickle-cell disease comprises a group of genetic disorders characterized by the inheritance of sickle haemoglobin from both parents. Sickle-cell disease carries a huge psycho-social burden which has impacts on the physical, psychological, social and occupational well-being as well as levels of independence on adolescents.

**Objective:**

To investigate the psycho-social problems of adolescents with sickle-cell anaemia in Ekiti State. The study also examined whether the variables of age and educational level would influence the psycho-social problems of adolescents with sickle-cell anaemia in Ekiti State.

**Methods:**

Descriptive survey design was adopted for the study. Purposive sampling technique was adopted to draw a total of 121 respondents. A questionnaire was used to collect data for the study. Mean and rank order was used to answer the research question while Analysis of Variance was used to test the hypotheses at 0.05 level of significance.

**Results:**

Findings revealed that psycho-social problems of adolescents with sickle-cell anaemia are limited in the choice of career, find it difficult to get suitable partner in marriage and SCD adolescents usually manifest emotional upset, misbehaviour, and have learning problems. Findings also revealed that there were significant differences in the psycho-social problems of adolescents with sickle-cell anaemia based on age and educational level.

**Conclusion:**

Majority of the respondents attested to the psycho-social problems facing adolescents with sickle-cell anaemia. Based on the findings of the study, it was recommended that social workers should be employed in health sectors; government should offered standard health care for all adolescents with sickle cell disease.

## Introduction

One particular disease that may affect a person's psychosocial adjustment is sickle-cell anaemia. During the early 1920's, V.R. Mason identified and described the term “sickle-cell anemia” as a disease in which one's red blood cells become distorted from their shapes^1^,^2^. In fact, sickle-cell disease stems from an inherited abnormality in hemoglobin, which consists of molecules in each cell that carry oxygen to all parts of the body. In the case of sickle-cell, the hemoglobin releases too much oxygen. Once oxygen is reduced enough, the cells become elongated or “sickled” red blood cells that clog the small blood vessels.

Sickle-cell disease comprises a group of genetic disorders characterized by the inheritance of sickle haemoglobin (Hb S) from both parents, or Hb S from one parent and a gene for abnormal haemoglobin from the other parent. The presence of HbS can cause red blood cells to change from their usual biconcave disc shape to a crescent or sickle shape during de-oxygenation. Upon re-oxygenation, the red cell initially resumes a normal configuration, but after repeated cycles of “sickling and un-sickling,” the erythrocyte becomes damaged permanently and may remain sickled or may hemolyze. This hemolysis is responsible for the anaemia that is the hallmark of sickle-cell disease. Complications may include painful episodes involving soft tissues and bones, acute chest syndrome, priapism, cerebral vascular accidents, and both splenic and renal dysfunction. Historically, common causes of mortality among children with sickle-cell disease include bacterial infections, splenic sequestration crisis and acute chest syndrome.

Sickle-cell disease is a chronic illness that affects more than 250 million people worldwide^3^. Sickle-cell disease is life-altering for most families. Learning to accept, cope and respond to this chronic illness requires that the counsellor and family work together. Cooperation occurs best in an environment where the family feels comfortable, safe and un-judged and the counsellor sets a tone for the relationship. That tone should encourage the family to view the counsellor as a resource, confidante and advocate. When working with children and families affected by sickle-cell disease, it is important to develop a comprehensive approach that encompasses psychosocial issues. Working to understand the issues faced by many of these families will help improve relationships and ensure a positive outcome.

Painless and painful crises are common phenomena in sickle-cell disorder which is a genetic blood disorder caused by the presence of an abnormal form of hemoglobin. It is an inherited autosomal recessive genetic disorder of hemoglobin (Hb) structure caused by point mutation at the sixth position in beta globin chain, valine substituting glutamic acid. Sickle-cell anemia (SCA) is a chronic disease and it would be expected that the children with SCA are at high risk of developing psychosocial problems^1^.The psychosocial theme takes into account the person's total life situation. This theme builds from an understanding of individual development, interpersonal influence, influence of significant others, and influence of significant environments and systems on the development and maintenance of healthy living^1^.

Nearly one third of Nigeria's total population is between the ages of 10 and 244. Nigerian adolescents' sizeable share of the population makes them integral to the country's social, political and economic development. Inadequacy of information, counselling and services appear to make young people vulnerable to sickle-cell disease. However, many organizations are working to improve adolescents' health life through advocacy and prevention programmes.

In fact, in many countries in Africa, especially Nigeria and particularly in Ekiti State, adolescents constitute approximately thirty-three per cent of the population5. Adolescence is the transition from the world of childhood to the world of adulthood. It is a period of physical and emotional development almost as rapid as the first decade of life. At this time, the body matures and the mind becomes more questioning and independent. It seems the physiological changes which take place during this period of transition from childhood to adulthood result in the formation and maturation of essential organs and present the first challenge to healthy adolescent growth.

Psychological complications in patients with SCD mainly result from the impact of pain and symptoms on their daily lives and society's attitudes towards them. Early research in psychological aspects of SCD examined the extent of its impact on both children and adults, and the functioning of affected families. Young people are perceived as generally healthy, and are not in need of special health services^6^. Sickle-cell adolescent health needs, behaviours and expectations are unique and routine health care services appear not well geared to provide these services. This is a huge challenge in a developing country like Nigeria due to various cultural and social barriers. In order to provide acceptable services with adequate utilization, in-depth exploration of social and cultural barriers and understanding the needs and expectations of sickle-cell adolescents counselling is a great necessity.

Counselling is needed wherever there are problems. The need and importance of counselling for adolescents with sickle-cell disease cannot be over-emphasized. Counseling for sickle cell is a process of basic education or giving information. It ought not to consist of giving advice. One major task of the sickle-cell counsellor is to be certain that the counsellee understands what sickle-cell trait is and what its implications for his family are. The other major task of the sickle-cell counsellor is to help individuals work through the psychosocial impact of being informed that they have a genetic condition. Counselling is most efficient when other educational techniques are employed concurrently. These may include pamphlets, brochures, cassettes, films, and other media. It is very desirable that sickle cell counselors give their counselees some concise written material on sickle cell trait and sickle cell anemia that they can take home and study at their leisure.

It is necessary for adolescent with sickle-cell anaemia to be properly counseled in order to cater for psycho-social problems facing them; hence, the study examined the psycho-social problems of adolescents with sickle-cell anaemia in Ekiti State.

## Research Question

This research question was raised based on the problem:

What are the psycho-social problems facing adolescents with sickle-cell anaemia in Ekiti State, Nigeria?

## Research Hypotheses

The following null hypotheses are postulated and tested for the conduct of the study:

There is no significant difference in the psycho-social problems of adolescents with sickle-cell anaemia based on age.

There is no significant difference in the psycho-social problems of adolescents with sickle-cell anaemia based on educational level.

## Methods

### Research Design

The research design that was used for this study is descriptive survey research design. The descriptive nature of this design makes it suitable for this study since it is aimed at investigating the psycho-social problems of adolescents' sickle-cell anaemia in Ekiti State, Nigeria.

### Participants and Setting

The population for the study comprised all adolescents with sickle-cell disease (SCD) in the sixteen Local Government Areas of Ekiti State. According to the statistics provided by Ekiti State ministry of Health^7^, the total number of Sickle-Cell Disease (SCD) adolescents in Ekiti State is approximately two thousand, three hundred and nine (2,309). The researcher adopted the purposive sampling method to select adolescent with SCD in selected General Hospitals in Ekiti State University Teaching Hospital (ESUTH) and Federal Teaching Hospital (FTH), Ido-Ekiti. Out of approximately two thousand, three hundred and nine (2,309) SCD patients attending the selected hospital in Ekiti State, an estimated sample size of 110 is made with an attrition rate of 10% (11) added to it, giving a total of 121 for the sample, in an agreement with Fishers formula less than 10,000.

### Instrumentation

The instrument used for the study is a researcher-designed questionnaire. The questionnaire has two sections, A and B. Section A sought to elicit the respondents' bio-data while section B contains 20 items that measures the psycho-social problems of adolescents with sickle-cell anaemia. The instrument was validated by experts in family medicine while the reliability of the questionnaire was determined through test re-test method with a co-efficient of 0.84.

### Ethical Issues

The objective of the research was clearly explained to the respondents by attaching informed consent forms to the questionnaire. Respondents were assured of utmost anonymity and confidentiality of any information provided on the questionnaire form. Each respondent's identity was protected as their names were requested.

### Data Analysis

The data generated were analyzed using mean and rank order. Hypotheses 1 and 2 were tested using Analysis of Variance (ANOVA). All the hypotheses were tested at 0.05 level of significance.

## Results

### Demographic Characteristics of the Participants

The characteristics of the participants by age shows that 72.7% (88) are between 13 and 15 years in age, 27.3% (27) are 16–17 years and 5.0% (6) are 18–19 years in age. This shows that respondents who are 13–15 years in age participated more in this study. The distribution of participants by educational level shows that 2.5% (3) participants have no formal education, 22.3% (27) participants have primary education, 68.6% (83) participants have secondary education and 6.6% (8) of the participants have tertiary education. This indicates that respondents who have secondary education participated more in the study.

### Research Question 1:

What are the psycho-social problems facing adolescent with sickle-cell anaemia in Ekiti State?

Table 1 presents the mean and rank order of respondents' expression on the psycho-social problems facing adolescents with sickle-cell anaemia. Item 9 was ranked 1st with mean score of 3.64 and states that “I am limited in the choice of career;”, item 10 ranked 2nd with mean score of 3.14 and states that “I find it difficult to get suitable partner in marriage” and item 5 was ranked 3rd with mean score of 3.11 and states that “SCD adolescent usually manifest emotional upset, misbehaviour, and have learning problems”. The table indicates that most of the items have the mean scores that are above the mid-cut off point of 2.50; this indicates that the respondents attested to the psycho-social problems facing adolescents with sickle-cell anaemia.

**Table 1:** Mean and Rank Order on the Respondents' Expression on psycho-social problems facing adolescent with sickle-cell anaemia

Item No.	Psycho-social problems facing adolescent with sickle-cell anaemia	Mean	Rank
9	I am limited in the choice of career	3.64	1st
10	I find it difficult to get suitable partner in marriage	3.14	2nd
5	SCD adolescent usually manifest emotional upset, misbehaviour, and have learning problems	3.11	3rd
1	People don't like to associate with me	3.07	4th
16	I am often respected by others	3.05	5th
6	I don't usually feel composed in the public	2.92	6th
14	I find it difficult to eat any type of food	2.88	7th
13	I feel irritated	2.85	8th
2	I'm usually scared and feel unprotected whenever am in the midst of friends	2.84	9th
19	My parents love me so much	2.74	10th
17	I tolerate others	2.73	11th
7	I have no sense of belonging	2.69	12th
11	I'm scared about my life span	2.60	13th
18	I am always happy with people	2.55	14th
15	I always feel honoured by the people	2.46	15th
12	My immediate family are unconcern about my welfare	2.36	16th
3	I usually experience pains in my body	2.29	17th
20	I get angry easily	2.14	18th
8	I do not have capacity for tedious work	2.07	19th
4	My physical appearance makes me uncomfortable	1.73	20th

### Hypotheses Testing

#### Hypothesis One:

There is no significant difference in the psycho-social problems of adolescents with sickle-cell anaemia based on age

Table 2 shows that calculated F-ratio of 5.18 is greater than the critical F-ratio of 3.00 with a corresponding p-value of .007 which is greater than 0.05 alpha level. Since the calculated F-ratio is greater than the critical F-ratio, the null hypothesis is therefore rejected.

Table 3 shows the DMRT indicating the significant difference noted in the ANOVA on Table 2. Group 1 with a mean score of 53.51 significantly differed from Group 2 with a mean score of 53.78, but significantly differed from Group 3 with a mean score of 58.83. All the groups differed from one another but the significant difference noted was as a result of the mean of Group 3 with the highest mean score, hence the significant difference noted in the ANOVA on Table 2 was brought about by respondents 18–19 years in age therefore, the hypothesis is rejected.

**Table 3:** Duncan Multiple Range Test (DMRT) Showing the Differences of the Respondents' Perception of the psycho-social problems adolescents with sickle-cell anaemia based on age

Duncan Groupings	N	Mean	Group	Age
A	88	53.51	1	13–15 years
B	27	53.78	2	16–17 years
C	6	58.83	3	18–19 years

**Table 2:** Analysis of Variance (ANOVA) showing the Respondents' Expression on psycho-social problems of adolescents with sickle-cell anaemia based on age

Source	df	SS	Mean Squares	Cal. F-ratio	Crit. F-ratio	p-value
Between Groups	2	159.206	79.603	5.18*	3.00	.007
Within Groups	118	1813.489	15.369			
Total	120	1972.694				

* Sig. at p < 0.05

#### Hypothesis Two:

There is no significant difference in the psycho-social problems of adolescents with sickle-cell anaemia based on educational level

Table 4 shows that calculated F-ratio of 5.76 is greater than the critical F-ratio of 2.60 with a corresponding p-value of .001 which is less than 0.05 alpha level. Since the calculated F-ratio is greater than the critical F-ratio, the null hypothesis is therefore rejected.

**Table 4:** Analysis of Variance (ANOVA) showing the Respondents' Expression on psycho-social problems of adolescents with sickle-cell anaemia based on educational level

Source	df	SS	Mean Squares	Cal. F-ratio	Crit. F-ratio	p-value
Between Groups	3	235.861	84.620	5.76*	2.60	.001
Within Groups	117	1718.833	14.691			
Total	120	1972.694				

* Sig. at p < 0.05

Table 5 shows the DMRT indicating the significant difference noted in the ANOVA on Table 4. Group 1 with a mean score of 55.67 significantly differed from Group 2 with a mean score of 54.89, but significantly differed from Group 3 with a mean score of 53.00 and group 4 with mean score of 58.25. All the groups differed from one another but the significant difference noted was as a result of the mean of Group 4 with the highest mean score, hence the significant difference noted in the ANOVA on Table 4 was brought about by respondents in tertiary education therefore, the hypothesis is rejected.

**Table 5:** Duncan Multiple Range Test (DMRT) Showing the Differences of the Respondents' Perception of the psycho-social problems adolescents with sickle-cell anaemia based on educational level

Duncan Groupings	N	Means	Group	Educational level
A	3	55.67	1	No Formal Education
B	27	54.89	2	Primary Education
C	83	53.00	3	Secondary Education
D	8	58.25	4	Tertiary Education

## Discussion

The findings of the study revealed the psycho-social problems of adolescents with sickle-cell anaemia are limited in the choice of career, find it difficult to get suitable partner in marriage and SCD adolescent usually manifest emotional upset, misbehaviour, and have learning problems. This was supported by Egbochukwu^2^ who stressed that one particular disease that may affect a person's psychosocial adjustment is sickle-cell anaemia. The physiological effects of the disease may be pain, shortness of breath, poor- health, constant colds, sore throats, jaundice, fatigue, and loss of appetite. Still some physical complications may appear for longer priods of time, such as surgical scars, dental deformities, impaired growth, leg ulcers, and increased susceptibility to infections, tissue damage to vital organs, and swollen hands and feet^8^. Some individuals appear to have an emotional tolerance to the disease. The persons with sickle-cell anaemia may experience particular feelings of frustration, anxiety, anger, resentment, and helplessness, occasioned by the prospect of being ill for the rest of their life or having a potentially fatal disease, feelings of defectiveness, fear, and pre-occupations with death.

Another finding revealed that there was a significant difference in the psycho-social problems of adolescents with sickle-cell anaemia based on age. In fact, in many countries in Africa, especially Nigeria and particularly in Ekiti State, adolescents constitute approximately thirty-three per cent of the population^5^. Adolescence is the transition from the world of childhood to the world of adulthood. It is a period of physical and emotional development almost as rapid as the first decade of life. At this time, the body matures and the mind becomes more questioning and independent. It seems the physiological changes which take place during this period of transition from childhood to adulthood result in the formation and maturation of essential organs and present the first challenge to healthy adolescent growth. Young people are perceived by World Health Organization6 as generally healthy, and are not in need of special health services. Sickle-cell adolescent health needs, behaviours and expectations are unique and routine health care services appear not well geared to provide these services. Adolescents with SCD often fear dying at a young age^9^,^10^. This fear has been attributed to the adolescent's experience of complications of SCD, neardeath experiences, or the death of a friend or peer with SCD. It is also possible that a common misconception that persons with sickle cell will die before reaching age 21 might have contributed or heightened the fear of death. It was evident that the support of family and friends played a pivotal role in helping these adolescents think positively about life.

Another finding also revealed that there was a significant difference in the psycho-social problems of adolescents with sickle-cell anaemia based on educational level. Moreover, persons with sickle-cell anemia experience problems in social relationships, due to consistent absence from school and physical complications, sickle-cell children are held back from normal grade advancement and have poor relationships with other children^3^. Adolescents with sickle-cell disease experience a longer period of conflict between dependency and independence. After reaching adulthood, frequent episodes of pain or disease-related problems may affect employment. Persons with sickle-cell may be stigmatized because of this disease. Some children with sickle-cell disease are frequenty absent from school^11^. These absences may be the result of a painful episode, hospitalization, out-patient visits and procedures or other illnesses. Frequent absences from school may result in incomplete class work and incomplete development of social skills. Students can feel disenfranchised from classroom activities and classmates^12^,^13^.

## Conclusion

The psycho-social problems of adolescents with sickle-cell anaemia are limited in the choice of career, find it difficult to get suitable partner in marriage and SCD adolescent usually manifest emotional upset, misbehaviour, and have learning problems. There were significant differences in the psycho-social problems of adolescents with sickle-cell anaemia based on age and educational level.

## Recommendations

Social workers should be employed in health sectors to assist adolescents with sickle-cell disease during clinic visits.

Counsellors should specially focus on adolescents with sickle-cell disease and device a means to encourage them cope in the school and society at large.
